# Temporal Dynamics and Spatial Patterns of *Aedes aegypti* Breeding Sites, in the Context of a Dengue Control Program in Tartagal (Salta Province, Argentina)

**DOI:** 10.1371/journal.pntd.0004621

**Published:** 2016-05-25

**Authors:** Manuel Espinosa, Diego Weinberg, Camilo H. Rotela, Francisco Polop, Marcelo Abril, Carlos Marcelo Scavuzzo

**Affiliations:** 1 Fundación Mundo Sano, Buenos Aires, Argentina; 2 Comisión Nacional de Actividades Espaciales, Falda del Carmen, Córdoba, Argentina; Centers for Disease Control and Prevention, Puerto Rico, UNITED STATES

## Abstract

**Background:**

Since 2009, Fundación Mundo Sano has implemented an *Aedes aegypti* Surveillance and Control Program in Tartagal city (Salta Province, Argentina). The purpose of this study was to analyze temporal dynamics of *Ae*. *aegypti* breeding sites spatial distribution, during five years of samplings, and the effect of control actions over vector population dynamics.

**Methodology/Principal Findings:**

Seasonal entomological (larval) samplings were conducted in 17,815 fixed sites in Tartagal urban area between 2009 and 2014. Based on information of breeding sites abundance, from satellite remote sensing data (RS), and by the use of Geographic Information Systems (GIS), spatial analysis (hotspots and cluster analysis) and predictive model (MaxEnt) were performed. Spatial analysis showed a distribution pattern with the highest breeding densities registered in city outskirts. The model indicated that 75% of *Ae*. *aegypti* distribution is explained by 3 variables: bare soil coverage percentage (44.9%), urbanization coverage percentage(13.5%) and water distribution (11.6%).

**Conclusions/Significance:**

This results have called attention to the way entomological field data and information from geospatial origin (RS/GIS) are used to infer scenarios which could then be applied in epidemiological surveillance programs and in the determination of dengue control strategies. Predictive maps development constructed with *Ae*. *aegypti* systematic spatiotemporal data, in Tartagal city, would allow public health workers to identify and target high-risk areas with appropriate and timely control measures. These tools could help decision-makers to improve health system responses and preventive measures related to vector control.

## Author Summary

As reported in Porcasi et al., in Argentina we are working on an integrated risk stratification system based in geospatial technologies that have moderately consolidated national scale, but need more understanding of its urban scale mechanisms. In this work, relevant results are shown on how *Ae*. *aegypti* breeding sites are distributed in dynamic spatial patterns in a small city on northern Argentina. 5 years of entomologic data were obtained by Mundo Sano Foundation, which is implementing an *Aedes aegypti* Surveillance and Control Program in Tartagal City (Salta Province, Argentina). The focus of this contribution is based on the difference that can found between one year data typical analysis and long term temporal evolution of spatial patterns. Although environmental sanitation activities and chemical control of breeding sites with larvicide were performed after each entomological surveillance all around Tartagal, sectors with higher densities of breeding sites remained present throughout study period. Nonetheless, the distribution of breeding sites showed a spatial dynamic with high density clusters in city outskirts.

## Introduction

Mosquitoes of *Aedes* genus are the principal vectors of Dengue, Yellow Fever, Chinkungunya and Zika viruses in the Americas [1,2]. *Aedes aegypti* (Diptera: Culicidae) transmits Dengue virus in the tropical and subtropical South America regions, and its transmission is influenced by various factors, including vector mosquito density, circulating virus serotypes, and human populations susceptibility [3]. In Argentina, *Ae*. *aegypti* is the most relevant mosquito from epidemiologic point of view. This specie is characterized by its adaptation to the urban environment, its capacity and preference of breeding in artificial containers [4], the resistance of its eggs to desiccation and the feeding behavior of the female which bites in multiple occasions during each gonadotrophic cycle [5]. These characteristics, together with this vector wide distribution in Northern Argentina, constitute fundamental factors that influence circulation and transmission of Dengue and other related viruses in the region [6].

After a successful vector eradication campaign, at national level, in the 70´s [7], the first outbreak of dengue in Argentina was documented in 1998. Since then, intermittent outbreaks of the disease, with variable incidence rates, were registered in an almost continuous manner in the center and northern regions of the country [8]. A major dengue outbreak reached subtropical regions of Argentina in 2009, affecting more than 25,900 people including localities such as Buenos Aires and Córdoba [9,10]; although the largest percentage (over 90%) corresponded to case reports from Chaco, Catamarca and Salta provinces [8]. In this last province, in Tartagal city, around 665 dengue cases were confirmed including the first fatal case of this disease to be ever registered in Argentina [8]. From 2010 to 2014, a total of 338 suspected cases were registered in this city, from which 56 cases were confirmed (Hospital Provincial J.D. Perón, Tartagal, personal communication).

Taking into account 2009 epidemiological situation, in October of that year, Mundo Sano initiated an *Ae*. *aegypti* surveillance and control program with the objective of reducing the risk of dengue transmission in the city of Tartagal, Since then, a permanent surveillance system of breeding sites and key infestation determinant factors involved in was implemented to generate a systematic information record of high epidemiological value.

Considering Dengue native transmission, cases introduction from Paraguay, Bolivia and Brazil, and the absence of an effective vaccine [11], the north region of Argentine needs continuous vector control programs applications. Traditional *Ae*. *aegypti* control measures include elimination (breeding sources reduction) or larval habitats chemical treatment to prevent adults production, and space spray insecticides application to reduce adult population densities [12]. In this sense, current control methods require a clear identification of the areas and the periods of mayor entomologic risk, as well as the identification of the viral propagation flow in a community [13].

Multiple environmental factors, including biophysical and social ones, constitute a complex web that determines the spread of vector-borne diseases [14]. In this sense, Ostfeld and collaborators [15] indicated that despite the complexity, an analysis of the variables linked to vectors distribution and the identification of dengue cases can be a useful tool to generate future spatial and temporal scenarios for dengue. Spatial analysis of health events contribute to early detect situations involving diseases transmission [15], while the detection of disease clusters allows the identification of nonrandom events and the possibility of inferring their epidemiological determinants [16]. Surveillance tools, such as incidence maps, have been used to enhance public health preparedness for dengue outbreaks by providing a visual aid for decision-making [17,18].

On the other hand, the use of satellite images in epidemiological analyses allows the identification of key environmental factors (temperature, rainfall and humidity) that influence the dynamic of the vectors, as well as their interactions [19,20]. Since the beginning of remote sensing (RS) technology, studies on vector-borne diseases have focused on identifying and mapping vector habitats [21], assessing environmental factors related to vector biology [22–24] and studying disease epidemiology [25,26]. Recent studies investigated the application of RS and spatial analysis techniques to identify and map landscape elements, that collectively define vector and human population dynamics related to disease transmission risk [27,28]. In addition, the development of increasingly sophisticated Geographic Information Systems (GIS) and RS has provided a new set of tools for public health professionals to monitor and respond to health challenges [29,30].

In this frame, Louis and collaborators [12] have detected a great diversity of both predictors and modeling approaches employed to create dengue risk maps through a systematic review and determined that the field of predictive dengue risk mapping is young and still evolving. In addition, different studies propose measures of prevention and control of *Ae*. *aegypti* for the elaboration of maps based on the results obtained from a bounded availability (space-time) of recorded data from both, field data and satellite imagery [31–33]. In this sense, an increase in the quality (amount and accuracy) of the field data used for the development of predictive maps will allow public health workers to identify areas of high risk for adequate control of the disease [19,34,35]. Despite the knowledge of *Ae*. *aegypti* biology and the use of monitoring tools, the precise detection of high density spots of vector breeding sites, as places of occurrence of the disease, remains poorly understood.

Therefore, the purpose of this study was to analyze 5 years space-time dynamics of *Ae*. *aegypti* breeding sites and control actions effect on its populations, in Tartagal City (Prov. of Salta, Argentina). We discuss the predictive capacity of *Ae*. *aegypti* spatial distribution models, generated through environmental variables and minimal field data. This models constructed for dengue surveillance based on entomological risk maps, are considered a step in the generation for vector control strategies.

## Materials and Methods

### Study area

**Fig 1:**
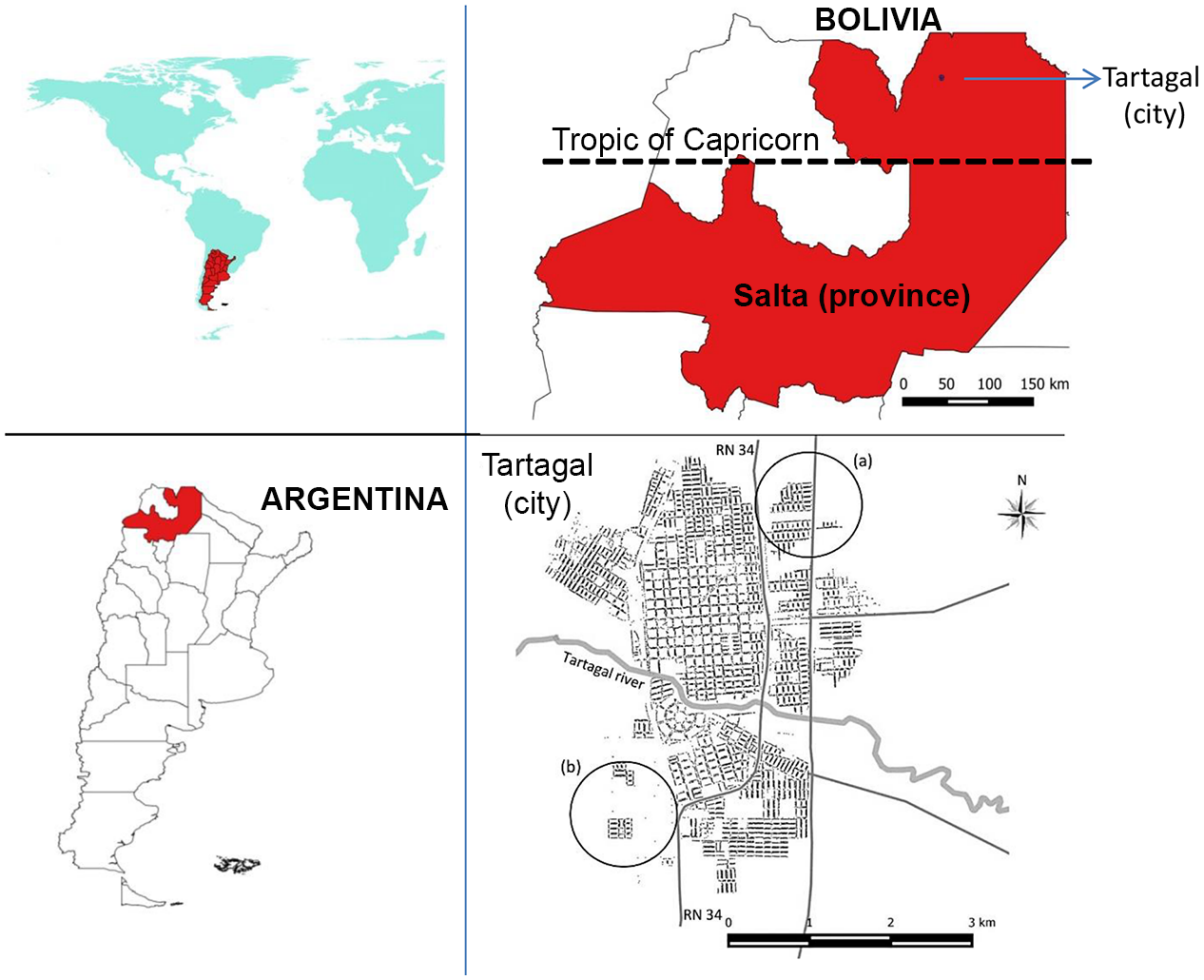
Study area. Left top, worldwide location. Left bottom, location of Salta province in Argentina. Right top, Salta-Bolivia border. Bottom right, detail of Tartagal city and neighborhoods included in the study, neighborhoods (a) Northeastern added in 2013 and (b) Southwest added in 2011.

Tartagal city is located at the base of the Argentinean sub-Andean hills (22°32’ S, 63°49’ W; 450 m above sea level) in Salta Province (Fig 1). As the third largest urban center of the province, with 79,900 inhabitants, it includes several ethnic groups such as native Amerindians. The city is located 100 km northern of Capricorn Tropic and to 55 km Southern of Argentinean-Bolivian border (Fig 1). The city is surrounded by subtropical native forests and crops such as beans, cotton, soybean, maize, grapefruit and tomato.

The climate is subtropical, with an average annual temperature about 23°C; and an average maximum of 39°C (in summer) and average minimum of 9°C a(in winter) respectively. Annual cumulative precipitation is about 1,100 mm, with a dry season from June to October with a monthly average rainfall of 30 mm, that sharply contrasts with the wet season from November to May with a monthly average rainfall reaching 160 mm.

Tartagal is characterized by a cultural diversity based on the presence of several autochthonous ethnic groups and emigrant population and continued migration from the bordering country of Bolivia. This feature produces an important effect on the cultural, social and economic profile of this community.

The urban area of Tartagal city covers approximately 15 km^2^, and is composed by 1,027 blocks and 17,911 housing units. Each of the housing units was georeferenced by the use of GPS receiver. In Fig 1, sectors (a) and (b) refer to new neighborhoods that were incorporated in data collection and entomological control actions performed in 2011 and 2013, respectively.

### *Ae*. *aegypti* data collection

Presence and abundance data of *Ae*. *aegypti* larval stages breeding sites were registered from 2009 to 2014 in Tartagal city, using a methodology called Focal Cycle (FC). This method consists in the entomological surveillance and chemical treatment of 100% of the housing units in the study area. A total of 10 FCs were performed; the first 8 were performed in a continuous manner between the years 2009 and 2012 (Table 1). The analysis of the data showed that during each year winter and spring, the presence of breeding sites and larval stages remained low. In order to optimize resources without losing any information, since 2012, during the winter-spring periods, random larval samplings were performed in 20% of the blocks of the city, alternating with FCs in the summer-autumn periods (Table 1). A total of 5 random larval samplings (denominated M1 to M5) were performed during the study period.

**Table 1:** Temporal sequence of entomological data collection during 2009–2014 period for Tartagal city.

Focal Cycle	Start	End	Year
**FC1**	12/05/2009	03/05/2010	2010*
**FC2**	03/08/2010	06/02/2010	2010*
**FC3**	06/02/2010	09/07/2010	2010
**FC4**	09/07/2010	12/21/2010	2010
**FC5**	01/03/2011	05/19/2011	2011*
**FC6**	05/18/2011	08/19/2011	2011
**FC7**	08/23/2011	12/16/2011	2011
**FC8**	12/19/2011	05/31/2012	2012*
**FC9**	10/05/2012	05/31/2013	2013*
**FC10**	11/22/2013	08/15/2014	2014*

FC (Focal Cycle);

*Focal Cycle performed during the summer-autumn period of each year and included in the data analysis.

During winter-spring period, chemical treatment was substituted by physical management and/or removal of containers that could serve as breeding sites. For each period, entomological field records consisted in the complete inspection of the housing units, within each block, registering information on the type and number of containers, grouped by the following categories: tires, tanks, drums, barrels, vases, pots, building materials, auto parts, bottles, cans, plastic, wells, cisterns, natural receptacles, and others (washing machines, refrigerators, toilets, etc.). Total number of containers was counted, such as the number of containers with water, and with larval stages. Larval stages were collected in individual tubes by container, labeled and transported to Mundo Sano entomological laboratory, in Tartagal city, for taxonomic determination using a specific morphological key [36].

Housing units were considered positive when they presented at least one container, with one or more larvae or pupae of *Ae*. *aegypti*. Additionally, a series of places were inspected and identified as critical breeding sites, since they presented an elevated number of containers in comparison to those registered in the housing units. The cemetery, municipal garbage dump, tire repair shops, small garbage accumulation sites and other similar places were included in this category.

In each FC, the entomological indexes at the housing unit level were calculated using the House Index (HI) = number of positive homes/number of houses inspected) x 100 and Breteau Index (BI) = total number of breeding sites/number of houses inspected) x 100 [37,38]. These indexes are generally accepted for operational use [39,40].

### Entomological control actions (chemical and environmental)

After the entomological data collection, focal control actions were performed in each housing unit which entailed, for each FC round, mechanical treatment (modification, elimination or destruction) together with the application of the larvicide in a 1 mg/L dose, following the guidelines elaborated by TDR/WHO and the Argentinian Ministry of Health [41,42]. These actions were accompanied by a communication campaign through the use of printed pamphlets destined to inform the general population about the disease and its risks. Moreover, with the objective of reducing the environmental burden of active and potential breeding sites generally accumulated in the peridomicile, neighborhood rallies were organized in collaboration with the local municipality and the participation of local public and private entities, to get rid of containers that favor the accumulation of water during the weeks prior to the start of the rainy season and during the summer months.

### Distribution and density of breeding sites of *Ae*. *aegypti* in Tartagal

In order to analyze the spatial and temporal distribution of the positive breeding sites, GIS vector layers were created including FCs data that were performed during summer and autumn of each year (Table 1). In this sense, and in order to comply with what was previously detailed, FC1 and 2 were combined since the interval of time between these FCs is equivalent to the time registered for the other FCs analyzed: FC5, FC8, FC9 and FC10 (Table 1). In order to avoid confusion, the unit of time of years will be used during the analysis to reference the FCs that correspond to the summer and autumn season of each year. Vector layers were generated using the free-access software Quantum GIS Desktop v2.6.1. Brighton (QGIS).

### Hot spots

Density breeding site maps were elaborated using discreet information (sites of individual sampling) through QGIS tool “heatmap”, in order to analyze the manner in which *Ae*. *aegypti* breeding sites were distributed in the city. Annual density breeding site maps (summer-autumn) were generated using the Kern density algorithm that calculates the density of positive points (grouping of the points) for a determined area. Using this methodology, the heat map allows for a visual identification of the hot spots for a particular time and place.

### Cluster analysis (SatScan)

The methodology of statistical spatial analysis exploration, developed by Kulldorf [43] was used to identify spatial clusters with *Ae*. *aegypti* larval stages presence, with greater density than those expected by a random distribution. The analysis would then indicate some areas with a greater presence of breeding sites than others. Sites with the presence of larval stages were indicated as positive cases (1) and those without any presence were indicated as negative controls (0).

The analysis consisted of a spatial scan through the superposition of exploratory circles, over sites with a record of larval presence. Each circle is a possible cluster and, taking into account the number of events inside and outside an expected number of events, each probability is calculated. The circle that presents the maximum probability, and an excess in the number of events observed versus expected, is defined as the most probable cluster [43]. In this case, the maximum size of the cluster was assigned as 30% of the total population under study.

In our analysis, for each place and window size (circle), the null hypothesis assumes that sites with the presence of larval stages are randomly distributed, while the alternative hypothesis indicates that there is a greater risk inside the window in comparison to the outside. A maximum of 999 Monte Carlo replications were performed in other to search for statistically significant composites. Only the composites that achieved statistical significance (p<0.05) under Bernoulli´s distribution were reported. The purely spatial exploration model was used for each year within 20102014 period in Tartagal. The statistical analysis was performed with SaTScan v9.3.1[44] software, while cartographic representations were done in QGIS software.

### Demographic and environmental characteristics of Tartagal

**Fig 2:**
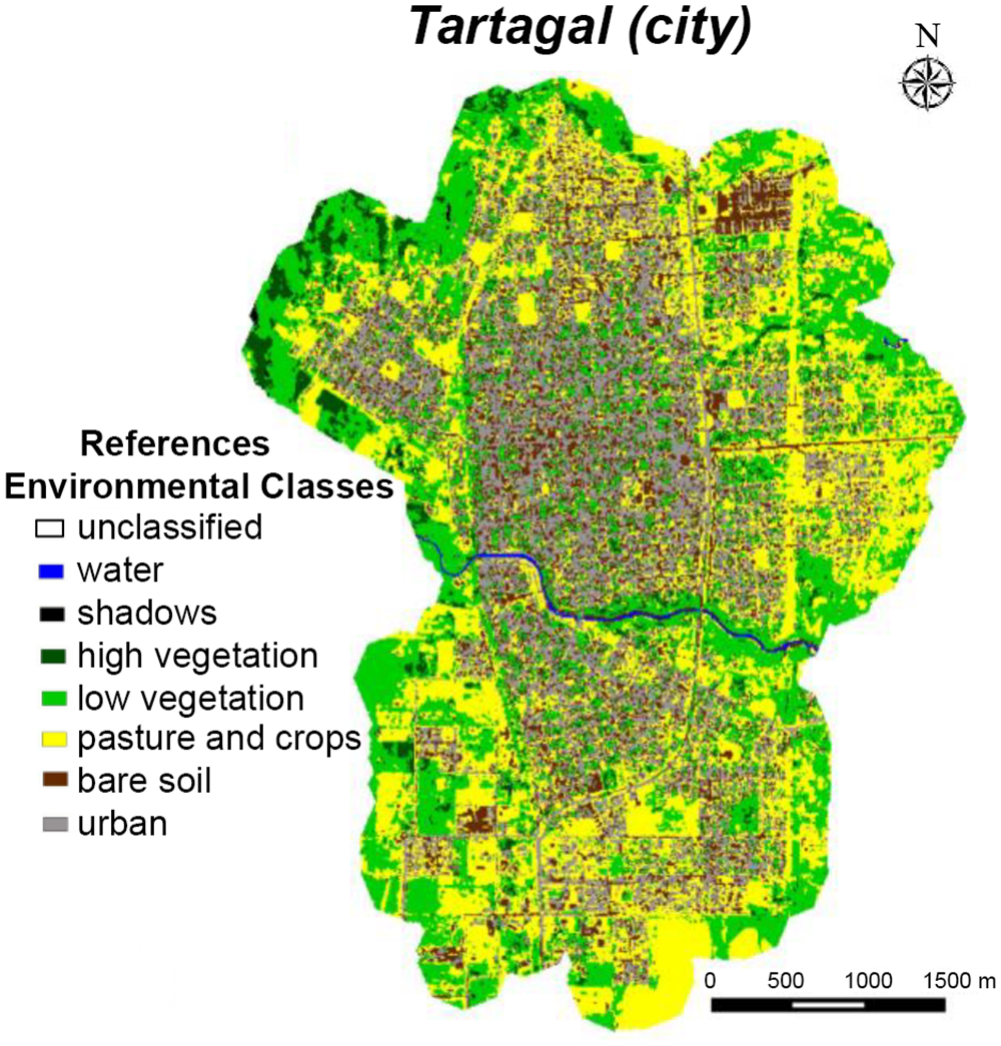
Land cover map of Tartagal city derived from an unsupervised classification (K-means) of SPOT 5 images, with seven different classes. Reference: unclassified (black), water (blue), shadows (black), high vegetation (dark green), low vegetation (light green), pasture and crops (yellow), bare soil (brown) and urban (grey).

SPOT images (Satellite Pour l’Observation de la Terre) were used to characterize the types of environmental coverage in Tartagal. This is a commercial high-resolution optical imaging Earth observation satellite system, operating from space. In this case, we used the SPOT 5 J product, of 10 meter resolution in multispectral mode, with four bands on short wave infrared: green (0.50–0.59 μm)–red (0.61–0.68 μm)–nearest infrared (0.78–0.89 μm) and middle infrared (1.58–1.75 μm). SPOT image (16-11-2013) data was used to generate land cover classifications and macro-environmental products of the study area (Fig 2). All the images used were supplied by the Comisión Nacional de Actividades Espaciales (CONAE).

### Land cover classification

Unsupervised classification (k-means) classifiers have been used to classify the image of the study area as described by Rotela and collaborators [45]. Seven land cover classes were identified: bare soil, low vegetation (grass), high vegetation (trees), urban buildings, superficial water, shadows, and pasture and crops (Fig 2). A set of ground truth points (about 35/40 points for each class) were generated using Google Earth in order to validate classification accuracy. The confusion matrix, when using control points, showed an overall accuracy of 79.4% and a Kappa coefficient of 0.74. The classes of (low and high) vegetation, and bare soil and pasture reached values above 70% accuracy, and the urban class presented lower registers. QGIS and ENVI 5.1[46] software were used to create the vectors and assess the accuracy of the classification.

### Environmental classes away from the classification

**Fig 3:**
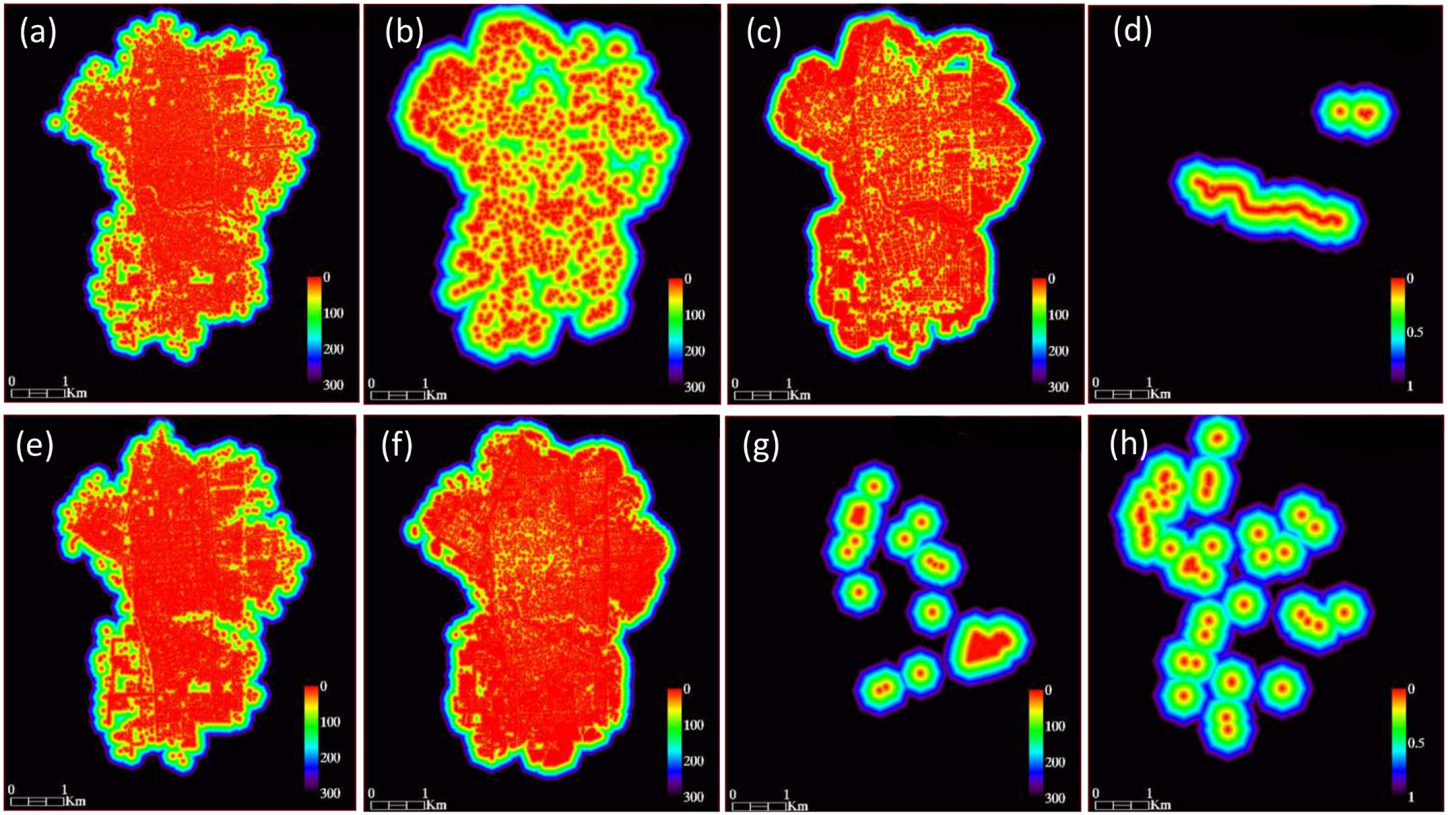
Distances environmental layers. References: a) bare soil, b) high vegetation, c) low vegetation, d) water, e) urban constructions, f) pasture, g) critical points, and h) shadows.

**Fig 4:**
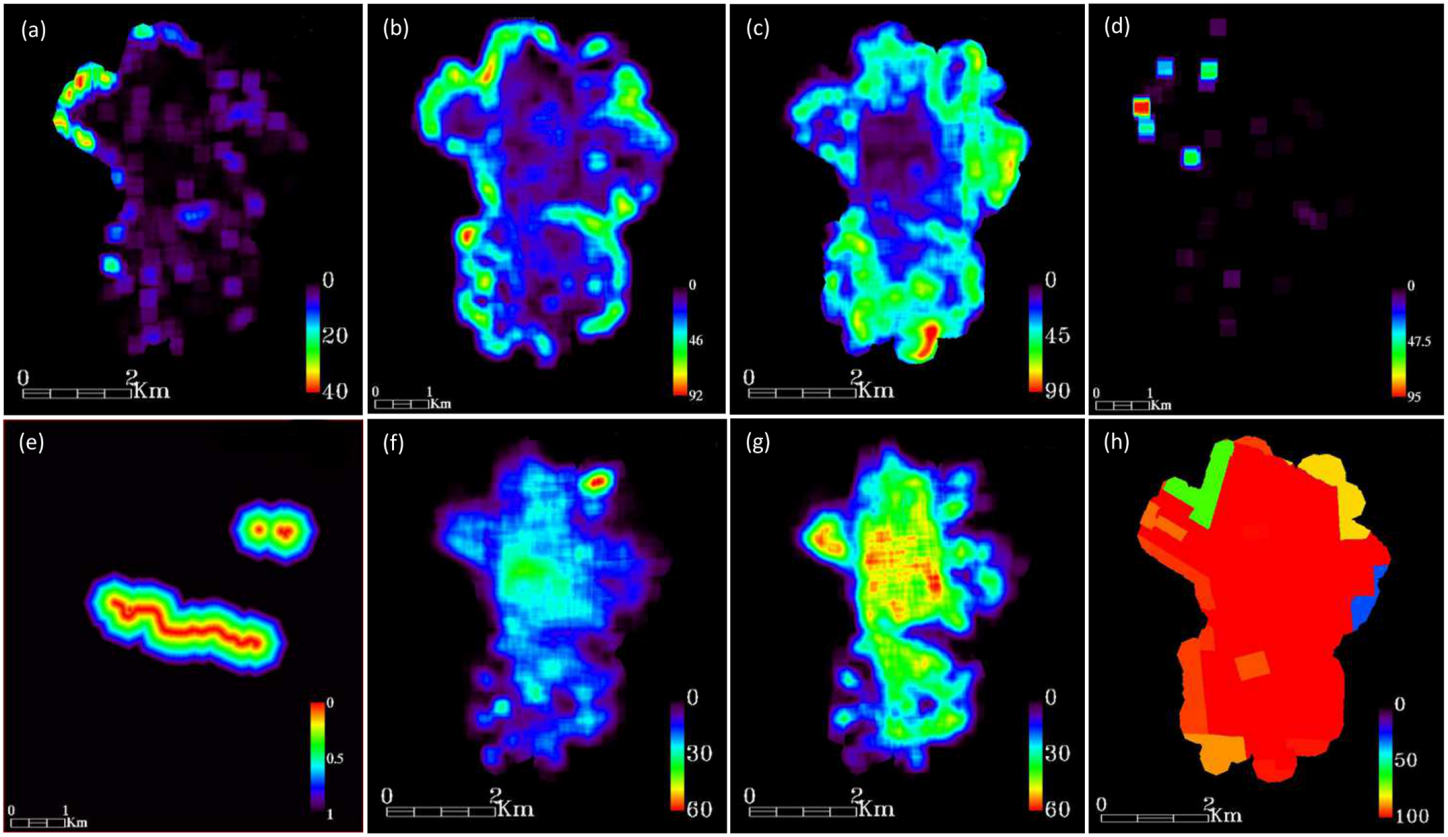
Percentage environmental layers. References: a) high vegetation, b) low vegetation, c) pasture, d) shadow, e) water, f) bare soil, g) urban constructions, and h) drinking water.

Based on the land cover classes previously created, two different types of macro-environmental variables were generated for each class, expressed as i) “distance maps or buffer image” (Fig 3) and ii) "percentage" of each land cover class (Fig 4), according to Rotela and collaborators [45]. In our study, the window size for the maps generated was 31x31 pixels, attributing to the central pixel the average value of the central window. A flight range of 150 m for *Ae*. *aegypti* [47,48] was used to generate the new land cover classes (distance and percentage), which could describe the environment that represents the average habitat of the species. All these analyses were performed using ENVI 5.1.

### Demographic classes

Tartagal information provided by the Instituto Nacional de Estadística y Censos (INDEC) was used to generate 2 layers that included demographic information related to the availability of drinking water (public network) (Fig 4h) and the distance to critical points (cemetery and garbage dump) (Fig 3g). First INDEC layer reflects the lack of this service, as an indicator of the use of containers for outdoor water storage, as potential generator of artificial *Ae*. *aegypti* breeding sites. INDEC information was transformed to a vector layer that included percentage values of the service per Census radio units by the use of QGIS software.

### Vector presence probability

In order to assess the contribution of each of the selected variables to the prediction model, MaxEnt software [49] was used to predict suitable sites for the development of *Ae*. *aegypti* breeding sites in Tartagal, based on the environmental requirements of the species [19,50–52]. Ecological modelling calculates the probability of vector breeding sites presence using environmental and demographic variables, and the actual vector breeding sites presence as training sites. All the positive sites for *Ae*. *aegypti* larval stages from the sampling performed in Tartagal during 2014 were used. In order to analyze the possible relationship of a product that compiled all variables, values are generated. Thus, each pixel of the study area presents a landscape value (indicated by the set of variables, see Table 2) and may have an associated value indicating the probability. This analysis is based on two basic premises, i) the first one relates to the presence of sites where the species successfully grow, and the second ii) refers to the selected environmental variables that adequately represent the ecological requirements of the species. Each presence site is indicated by a pair of geographic coordinates (WGS84 Datum), and represents a place where *Ae*. *aegypti* breeding sites were found during the sampling period. The Maximum Entropy approach (MAXENT) was used to model and predict the ecological niche distribution of the vector. In general, this algorithm detects non-random relationships between two data sets: i) georeferenced records of the presence of the species, and ii) a set of land cover type "raster", digital data representing the environmental and demographic variables relevant to determine the distribution of the species in a particular scale of analysis [49]. The environmental data set consists of 19 variables in raster format, of 10 m pixel size (see Table 2 for data access to Tartagal). For the generation of vector presence probability maps, we applied the Maximum Entropy method based on the MaxEnt 3.3.3a software [49], available online at http://www.cs.princeton.edu/~schapire/maxent/, reserving 25% of *Aedes aegypti* presence points for validation, and with a 1000 repetitions run.

**Table 2:** Environmental, climatic and demographic variables used to create *Aedes aegypti* distribution models for Tartagal.

Variable	Type
**1. Water supply (INDEC, 2010)**	**Demography**
**2. Hot points distribution**	**Demography**
**3. Urban distribution**	**Environmental**
**4. Urban coverage Percentage**	**Environmental**
**5. Bare soil distribution**	**Environmental**
**6. Bare soil coverage percentage**	**Environmental**
**7. Low vegetation distribution**	**Environmental**
**8. Low vegetation coverage percentage**	**Environmental**
**9. High vegetation distribution**	**Environmental**
**10. High vegetation coverage percentage**	**Environmental**
**11. Pasture distribution**	**Environmental**
**12. Pasture coverage percentage**	**Environmental**
**13. Wetland distribution**	**Environmental**
**14. Wetland coverage percentage**	**Environmental**
**15. Water distribution**	**Environmental**
**16. Water coverage percentage**	**Environmental**
**17. Land cover classes**	**Environmental**
**18. High NBRT values coverage percentage**	**Climatic**
**19. Nbrt_temperature distribution**	**Climatic**
**20. SPOT 5 Swir band**	**Environmental**
**21. POT 5 Xs1 band**	**Environmental**
**22. SPOT 5 Xs2 band**	**Environmental**
**23. SPOT 5 Xs3 band**	**Environmental**

Swir (short wave infrared, 1.58–1.75 μm); Xs1 SPOT (green, 0.50–0.59 μm); Xs2 SPOT (red, 0.61–0.68 μm); and Xs3 SPOT (near infrared, 0.78–0.89 μm); High NBRT values coverage percentage (coverage of Normalized Burn Ratio Thermal values bigger than Mean area value plus 1 Standard deviation).

## Results

### Entomological data collection of *Ae*. *aegypti*

**Fig 5:**
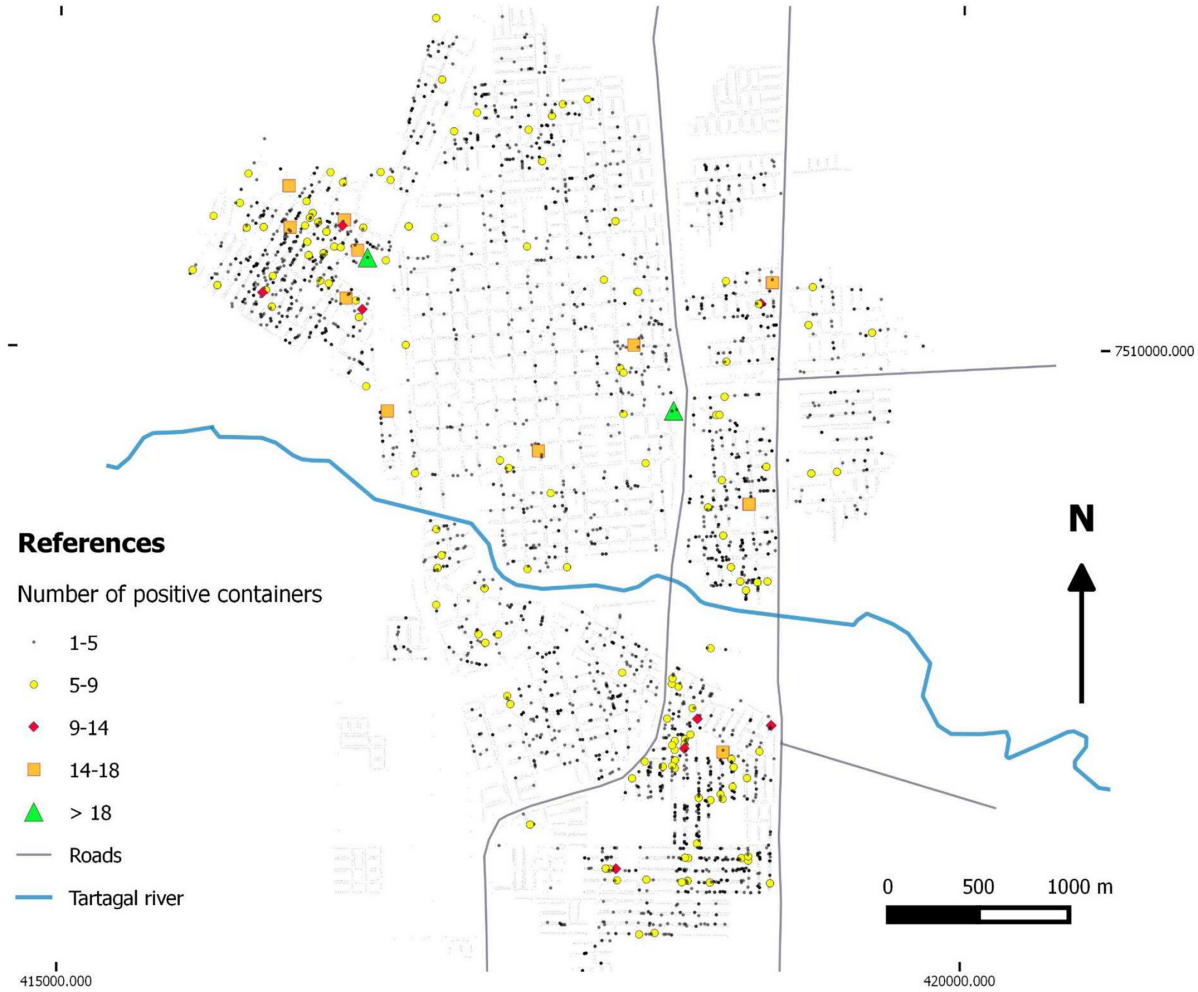
Distribution and total number of containers with *Ae*. *aegypti* larvae for all sites that were positive during the study period in Tartagal (2009–2014).

**Fig 6:**
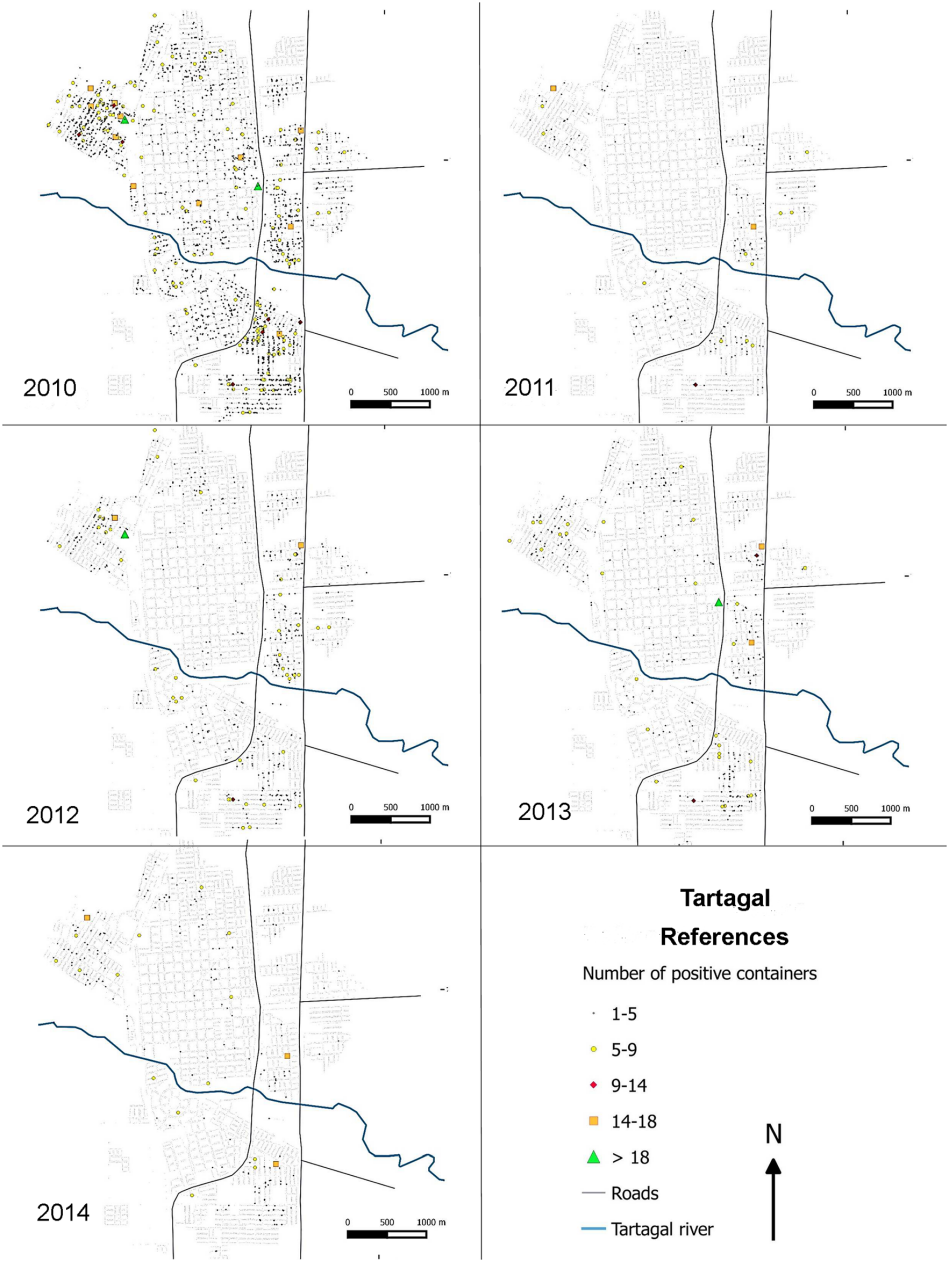
Distribution and total number of containers with larvae of *Ae*. *aegypti* for sites that were positive in Tartagal in the summer-autumn of each year (2009–2014).

The temporal variation of *Ae*. *aegypti* spatial distribution of positive breeding sites presented a wide distribution all around Tartagal city(Figs 5 and 6), with the highest densities spatially concentrated in city outskirts, in comparison to the central areas (Fig 5). The temporal representation of positive sites in the city registered variations in distribution and number over the years, observing a similar spatial pattern as previously described (Fig 6).

**Fig 7:**
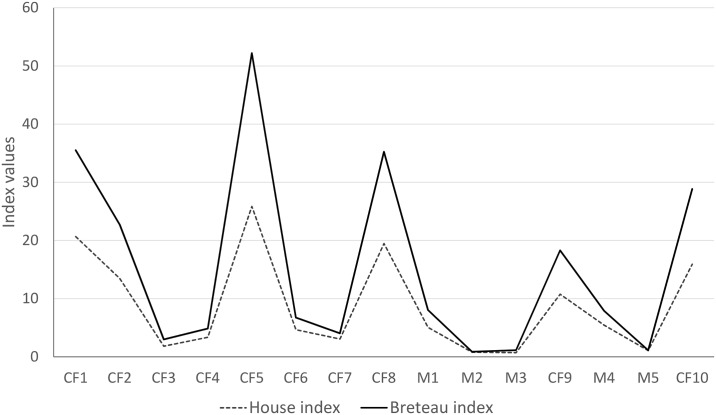
Fluctuation of the Stegomyia indexes (HI and BI) during the study sample period in Tartagal. References: FC (Focal Cycle); M (random sampling).

**Fig 8:**
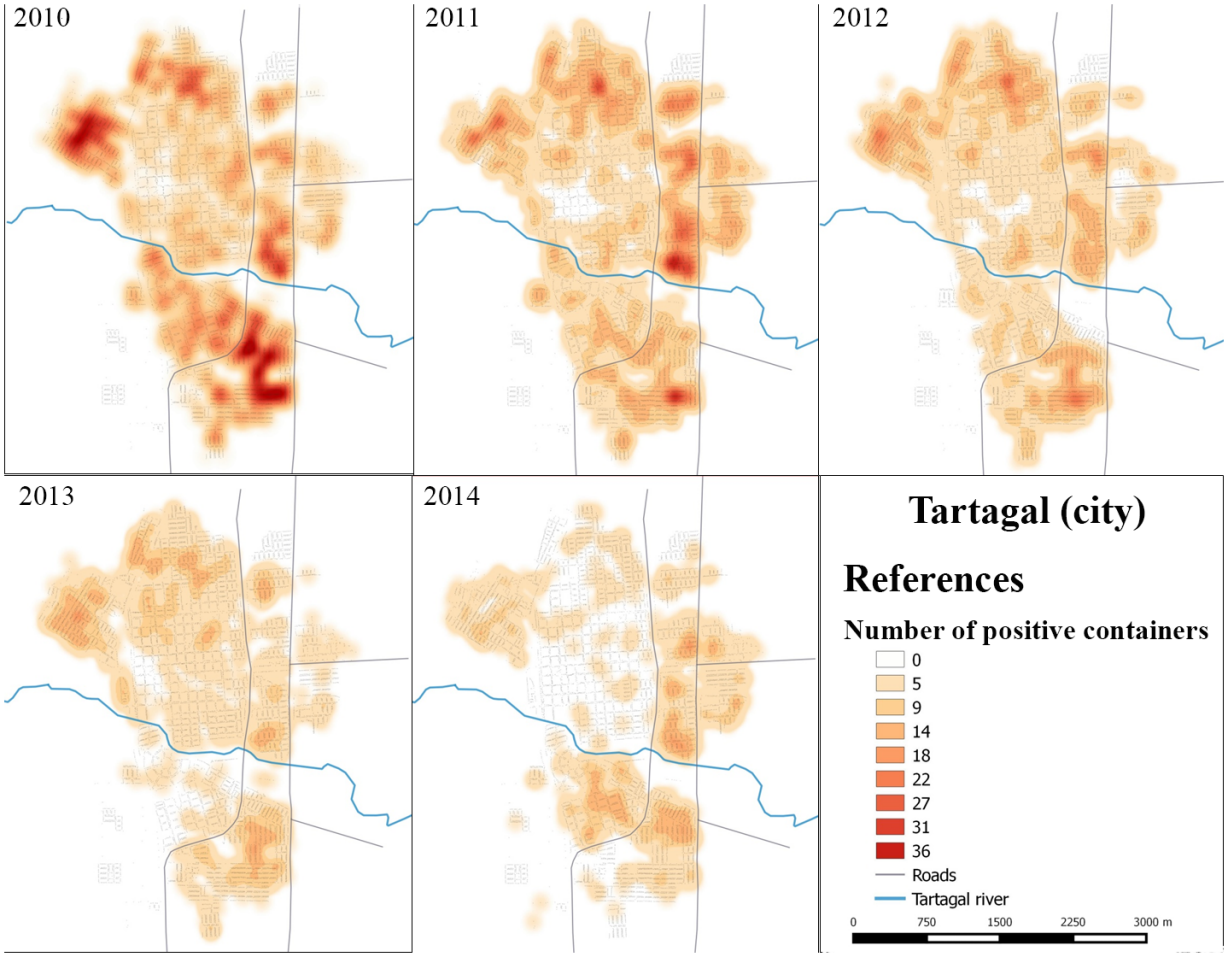
Annual density (summer-autumn period) of *Ae*. *aegypti* breeding sites for Tartagal.

Fig 7 shows that house indexes (HI and BI) values decreased between 2010 and 2014. Throughout this period, both indexes registered their highest levels during the summer and autumn seasons, which coincide with FC1, FC2, FC5, FC8, FC9 and FC10 (Table 1), while the lower values were associated with winter and spring (FC3, FC4, FC6, FC7, M1, M2, M3, M4 and M5) (Table 1 and Fig 8).

### Hotspots

Sectors with presence of *Ae*. *aegypti* breeding sites were distributed throughout the entire study area, especially at the beginning of the program during 2010 and 2011 (Fig 8). Through the analysis of hotspots, three important aspects were detected: 1) the gradual reduction in the density of breeding sites detected each year, 2) the presence of sectors with a density of breeding sites that persist throughout the study period analyzed, located in the northeast, north, east and southeast regions of Tartagal, and 3) the highest density of breeding sites were associated with peripheral sectors while the lowest ones were registered in the central areas of the city (Fig 8).

In 2010, the northeast and southeast Tartagal sectors reached the highest density of breeding sites, with up to 20 positive breeding sites per housing unit. These two sectors remained positive throughout the study period although with variations in the density values (Fig 8). During 2011 and 2012, the north and east sectors of the city were identified as areas with high density of breeding sites (Fig 8). The year 2013 showed a spatial configuration that was similar to the previous years but with a marked reduction in breeding site density (Fig 8), while in 2014, the areas that registered the highest density of breeding sites were sectors located in the east and southeast (Fig 8).

### Cluster analysis (SatScan)

**Fig 9:**
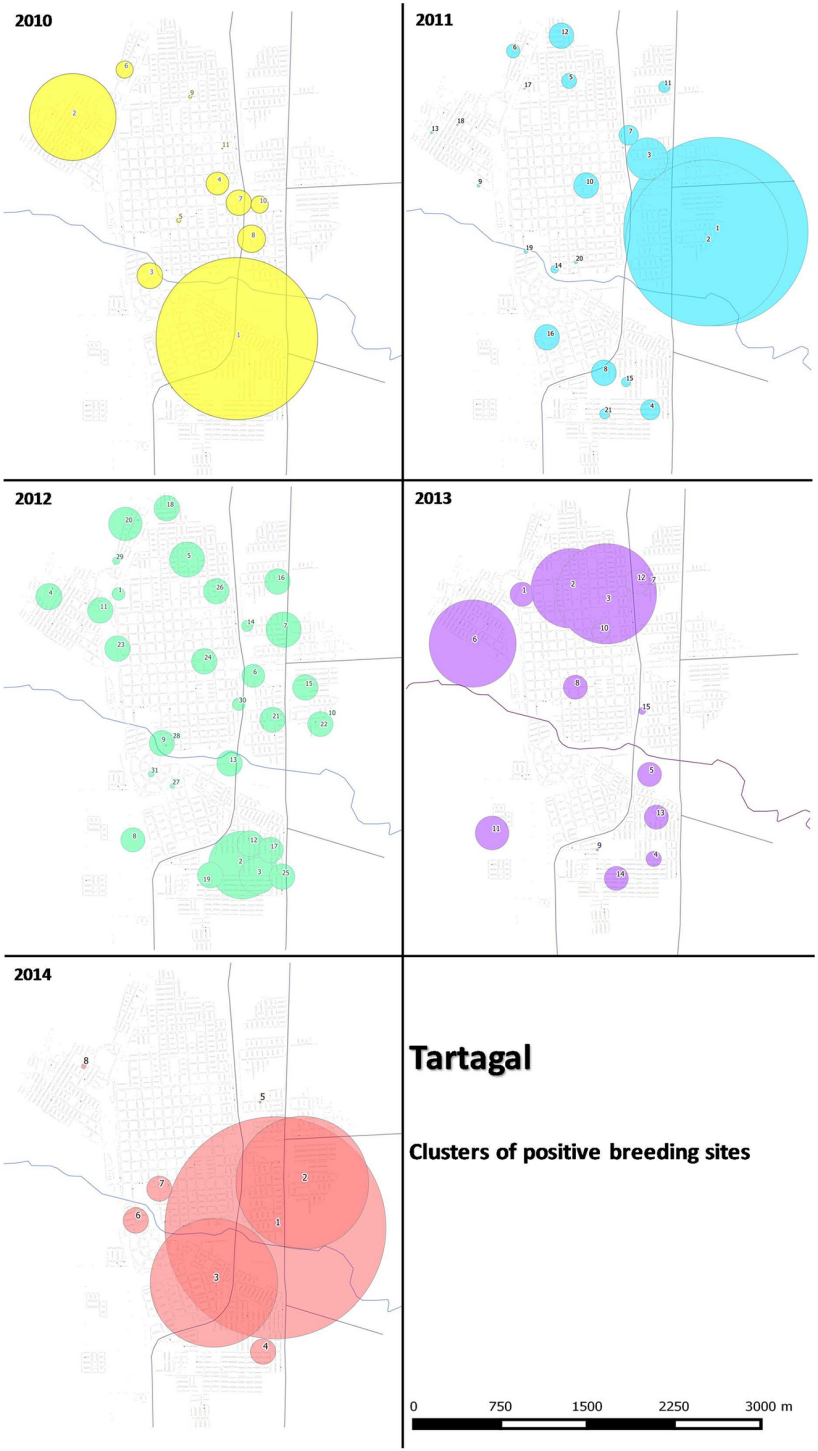
Main space-time clusters in Tartagal city. The different colored circles represent different years included in the analysis.

Statistically significant (p < 0.05) differences were observed in spatial clusters between 2010 and 2014 (Fig 9). In general, the clusters with the largest dimensions were located in the northeast, north, east and southeast sectors of the city, with clusters that varied in size throughout the study period (Fig 9). In 2011 and 2014, the east sector registered the highest clusters with radius greater than 1.5 km (Fig 9). On the other hand, in 2010, 2012 and 2014 the southeast sector presents clusters with radius greater than 0.5 km. In the northeast sector, clusters with radius greater than 0.5 km were registered only during 2010 and 2013, while in the north sector these size clusters were only registered in 2013 (Fig 9). Another aspect observed using cluster analysis was the concentration of clusters in one or two sectors of the city for the year 2010 and 2014. For the rest of the years, numerous clusters of radius size between 100 and 300 m were registered throughout different sectors (Fig 9).

### Ecological niche model

**Fig 10:**
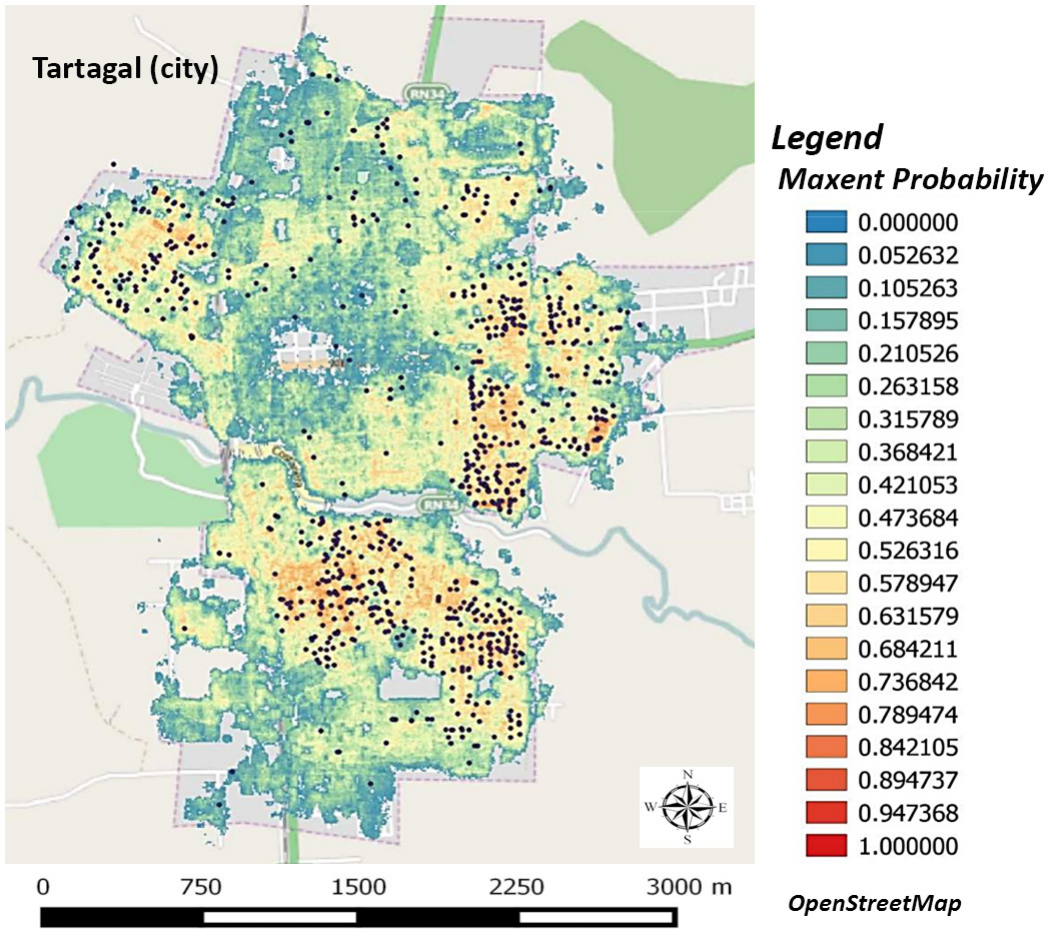
Probability map for *Ae*. *aegypti* breeding sites generated by an ecological niche model. The blue areas represent null risk of breeding sites and red areas represent the most suitable habitats for vector breeding sites.

The predictive map obtained by the model (Fig 10) was assessed with measurement accuracy (MaxEnt software), therefore the area under the curve (AUC) in receiver operating characteristic (ROC) analysis was scored at 0.918. Its predictive ability for the 2014 data set is classified as acceptable according to Parolo and collaborators [53]. The model predicted that the environmental variables that best explain 75% of the distribution of *Ae*. *aegypti* breeding sites were: the percentage of bare soil (44,9%), percentage of urbanization (13,5%), and water distribution (11,6%) (Table 3).

**Table 3:** Relative contribution of each environmental variable (contributions larger than 1%) to the Maxent model.

Variable	Percent contribution	Permutation importance
Percentage of bare soil	44.9	13.7
Percentage urban	13.5	8
Water distribution	11.6	19.1
Nbrt_temp	5.7	1.1
Percentage for pasture	5.2	11
Indec_percentage water	4.7	4.1
Bare soil distribution	4.3	4
Urban distribution	1.7	2.7
Hot points distributions	1.3	6.6
Percentage of low vegetation	1.2	3.9
Swir SPOT	1	0

Percent contribution: in each iteration of the training algorithm, the increase in regularized gain is added to the contribution of the corresponding variable. Permutation importance: for each environmental variable in turn, the values of that variable on training presence and background data are randomly permuted.

## Discussion

In this study we have presented relevant results on the spatial pattern dynamics of *Ae*. *aegypti* breeding sites, in a small city in the north of Argentina (Tartagal). Although environmental sanitation activities and breeding sites chemical control, with larvicide, were performed after each entomological surveillance in the city, the sectors with higher densities of breeding sites remained present throughout the study period. Spatial analysis (Figs 8 and 9) and predictive risk map (Fig 10) results, allowed us to indicate, with some confidence, the difficulty of implementing control measures and dengue surveillance in urban areas such as Tartagal. The wide spatial data set used in the present study, to generate the predictive habitat quality map, enabled us to register that, although the predictors used to adjust the model explained 75% of the distribution of positive breeding larvae sites, they failed to indicate other areas containing breeding sites with high density values (Fig 9).

The spatial pattern of a dengue epidemics would be determined by multiple synergic factors that occur concurrently in the environment, including entomological, demographic and epidemiological factors, among others [6]. Consequently, one of the key aspects to reduce the abundance of the vector is associated with the identification of the breeding sites where *Ae*. *aegypti* lays its eggs which later develop into larvae [11]. The density (hotspots) and distribution maps of the breeding sites in Tartagal indicated a greater proportion of breeding sites in the outskirts of the city (Figs 5, 6 and 8). These areas are characterized by a deficit in the potable water supply, especially during the summer months, which consequently promotes the accumulation of a diverse array of containers for water storage in the peridomicile. Generally, most of these containers are maintained uncovered, constituting excellent breeding sites for *Ae*. *aegypti*. A previous study conducted in the city of Clorinda, in the neighboring Province of Formosa, where this phenomenon was also observed, concluded that the practices related to water accumulation are due to cultural patterns adopted to face the lack of access and availability of this critical element, constituting a complex set of factors that influence the abundance of breeding sites and the population dynamics of *Ae*. *aegypti*, jeopardizing the efficacy of vector control programs [54]. This availability of breeding sites, with higher and lower densities of larval stages, suggest a subsequent uneven abundance and distribution of adults, as was already seen in studies conducted in Colombia [31].

Analyses performed allowed the identification of breeding sites hotspots in specific sectors of the city (Figs 8, 9 and 10). This scenario allows us to assume a spatial pattern of vector presence, with areas that the favor the presence and abundance of *Ae*. *aegypti*. During the Dengue epidemic on 2004 in Tartagal, a study detected spatial groupings of confirmed cases of dengue [42], which showed that dengue cases were concentrated in city outskirts, in agreement with *Ae*. *aegypti* breeding sites distribution observed in the current study (Figs 8, 9 and 10). Therefore, since the presence of dengue cases and positive breeding sites coincide in space, surveillance and control activities based on vector data would allow the identification of low and high risk areas for dengue transmission, allowing the planning of activities according to risk probability. Other studies have already suggested that an increase in vector density could lead to an increase in the vector-human contact which would translate into a higher viral transmission rate among the population [55,56]. Therefore, the detection of changes in vector density presents itself as an important factor in the epidemiology of the disease [31]. Taking into consideration *Ae*. *aegypti* urban characteristic, directly associated with the presence of larvae and the number and type of containers suitable for larval development [56], a greater proportion of breeding sites supposes a greater abundance of mosquitoes which leads to an increased probability of bites. In this scenario, the inferred spatiotemporal relationship between dengue cases [19] and breeding sites detected in our study could offer information on the presence of hotspots of *Ae*. *aegypti* infestation in Tartagal, which are regularly maintained in space and time.

During this study, after each round of entomological surveillance, activities of physical and chemical (application of larvicide) control of breeding sites were performed, diminishing the availability of suitable breeding sites and therefore impacting on the level of viral transmission [12]. From 2009 to 2014, a reduction of Stegomyia indexes (both HI and BI) was observed, which would be associated with a lower density of positive breeding sites (Figs 7 and 8). This tendency would suppose a positive effect of the control activities performed in Tartagal during this period which resulted in a decreased number of registered breeding sites (Fig 8). Nonetheless, the distribution of the breeding sites showed a spatial dynamic with high densities in the outskirts of the city, at the same time presenting sectors were high values were always maintained and others that varied throughout the years (Figs 8 and 9). This observation could be associated with the different conducts with respect to the use and management of water, since the lack of piped water in the housing units forces inhabitants to store water in different types of containers. At the same time, there are different behaviors with respect to containers care that, together with eco-environmental characteristics (temperature, land cover, etc.), constitute favorable scenarios for the development of the mosquito.

The relationship between environmental and climatic conditions, and *Ae*. *aegypti* dynamic, is well known and has been shown to affect the abundance and distribution of the mosquito [32,57–61]. Regardless the complexity of variables that affect the distribution of anthropophilic vectors, such as *Ae*. *aegypti*, tools for spatial analysis and GIS applied in the current study allowed us to find spatial relations between the positive breeding sites and the variables derived from remote sensing satellite (SPOT 5), which confirmed the urban characteristic of the vector. The aptitude model generated for Tartagal (Fig 10) showed that the presence predictive power of *Ae*. *aegypti* breeding sites for test data was acceptable, in accordance with Parolo and collaborators [53]. The distribution map showed that the higher probabilities of vector presence, with values that could exceed 70%, would be associated with three sectors in the city: one located in the south, one in the northwest and one in the east. The three sectors identified as the most likely to have vector presence (*Ae*. *aegypti* breeding sites) are isolated and separated by sectors that show a low probability (0–30%), and best explained by variables that represent typical urban patterns (bare soil, urbanization and distance to water) (Table 3). In this regard, it is important to highlight the usefulness of the HRG Spot 5 images for the urban characteristics associated to the presence of positive vector breeding sites.

Although the model was acceptable, it had a limitation in the north sector of Tartagal, which is not indicated a high infestation area, even though it presented a high suitability for the occurrence of positive sites, according to density (Fig 7) and hotspots (Fig 8) analysis. This inconsistency allows us to question the suitability of our model. As seen in Fig 8, the aptitude model showed a distribution similar to the one registered in the hotspot map of positive sites for the year 2014, therefore, the model is reflecting a particular situation of the distribution of positive breeding sites. This means that the use of a single period of entomological data for the elaboration of the model raises certain concerns in the moment of thinking of a comprehensive tool for the prevention of the disease, highlighting the importance of implementing sustainable programs for the collection of data and the elaboration of risk maps, in order to be able to tailor vector control actions adapted to the local reality.

Cities supply most of the habitat characteristics required by *Ae*. *aegypti* [62]. Recent studies [45], in accordance with our own field data, indicate a direct association between the presence larvae and the number of containers suitable for larval development, which would derive in an increased risk of mosquito larval infestation as well as the presence of adults in a particular area [12]. In Tartagal, and in accordance with other studies [15,63,64], the information from remote sensing has been used with the objective to provide information about the type of land cover that would allow us to indirectly estimate conditions favorable for the presence of breeding sites and survival of the mosquito. Although the aptitude levels obtained in our model do not include sites which we could consider as high risk, it suggests a lack of precision in the model predictability. This is in agreement with what has previously been indicated [18], which is that it is not easy to find precise spatial information on dengue useful for modeling. This calls attention to the manner in which the information provided is used. The great quantity of field data collected and analyzed in Tartagal allows us to evaluate the predictive capacity of the model. Therefore, we agree with Lois and collaborators [18] in that the predictive models must use a large time series of local data (entomological and environmental), which would allow to model different scenarios to assess the risk of the temporary effect on the predictions to generate more efficient control actions.

The results presented in this study show how entomological information and geospatial data (RS / GIS) could be used to infer scenarios which could then be applied in epidemiological surveillance programs and strategies for dengue control. But on the other hand, we can obtain unreliable scenarios when using insufficient (in quality / spatial and temporal coverage) input data, as shown in this work (fig 10), using only one year sampling data. The considerations presented in our study could be used by those who develop predictive maps and by public health workers to identify and target high-risk areas for dengue transmission. The approaches generated in this study could contribute information to decision-makers that could improve health system responses and prevention measures related to vector control.

### Future directions

Population dynamics of *Ae*. *aegypti* observed in our study, during 5 years of continuous work, allowed us to evaluate and fine tune our control strategy in the local context. Therefore, in the future, we would have to consider certain variables not currently contemplated: 1) determine the periodicity of control actions in accordance to the operational capacity of the work groups, 2) provide a solution for closed or uncooperative housing units which escape control activities and constitute sources of re-infestation, and finally, 3) determine the volume of data necessary for the elaboration of a highly predictive model for dengue transmission.
